# Brückenvenenverletzungen bei Schütteltrauma: Forensisch-radiologische Metaanalyse unter besonderer Berücksichtigung des „Tadpole“-Zeichens

**DOI:** 10.1007/s00117-020-00780-5

**Published:** 2020-12-08

**Authors:** D. Wittschieber, H. Muggenthaler, G. Mall, H.-J. Mentzel

**Affiliations:** 1 Institut für Rechtsmedizin, Universitätsklinikum Jena, Friedrich-Schiller-Universität Jena, Am Klinikum 1, 07747 Jena, Deutschland; 2 Institut für Diagnostische und Interventionelle Radiologie, Sektion Kinderradiologie, Universitätsklinikum Jena, Friedrich-Schiller-Universität Jena, Jena, Deutschland

**Keywords:** Misshandlungsbedingtes Kopftrauma, Kindesmisshandlung, Nichtakzidentelle Kopfverletzung, Subduralhämatom, Forensische Radiologie, Abusive head trauma, Child abuse, Non-accidental head injury, Subdural hematoma, Forensic radiology

## Abstract

Das Schütteltrauma-Syndrom ist eine häufige Variante misshandlungsbedingter Kopfverletzungen bei Säuglingen und Kleinkindern und nach wie vor Gegenstand intensiver Forschungsbemühungen. Unter Verwendung verschiedener Bildgebungsmodalitäten wurden in den letzten Jahren mehrere Studien zur diagnostischen und forensischen Relevanz traumatisierter Brückenvenen durchgeführt. Im vorliegenden Beitrag werden der aktuelle Forschungsstand dargestellt und forensische Implikationen erörtert. Im Ergebnis der Metaanalyse der sieben gegenwärtig vorliegenden Untersuchungen ist festzustellen, dass Brückenvenenverletzungen bzw. Brückenvenenthrombosen häufig als rundlich und erweitert wirkende bzw. tubulär gestaltete Strukturen imponieren. Das „Tadpole“-Zeichen kann hierfür als ein wertvolles Hilfsmittel zu deren Identifizierung dienen. Insbesondere T2*/SWI-Sequenzen ermöglichen eine gute Detektierbarkeit dieser Läsionen und sollten bei Verdacht auf eine misshandlungsbedingte Kopfverletzung immer zusätzlich erstellt werden. Schlussfolgernd ist zu empfehlen, dass das Vorhandensein von radiologisch detektierbaren Brückenvenenverletzungen stets Anlass dazu geben sollte, auch nach weiteren Anzeichen einer Kindesmisshandlung zu suchen.

## Abstract (en)

Shaken baby syndrome is a common variant of the abusive head trauma in infants and toddlers and is still subject of intensive research. In recent years, a number of radiological studies on the diagnostic and forensic relevance of injured bridging veins were conducted using different imaging modalities. The present article will give an overview on the current state of research in this field and will discuss the forensic implications. The meta-analysis of the seven currently existing studies revealed that injuries of the bridging veins and bridging vein thromboses, respectively, frequently appear as rounded, enlarged, and/or tubular structures. The “tadpole sign” may serve as a valuable tool for the identification of these formations. Especially, T2*/SWI (susceptibility-weighted imaging) sequences allow for good detectability of these lesions and should always be generated when abusive head trauma is suspected. In conclusion, it can be recommended that the presence of radiologically detectable bridging vein injuries should give reason to search for other manifestations of physical child abuse.

Die sichere Feststellung eines Schütteltrauma-Syndroms („shaken baby syndrome“, SBS), einer häufigen Variante des misshandlungsbedingten Kopftraumas („abusive head trauma“, AHT), stellt noch immer eine große interdisziplinäre Herausforderung dar. Sie ist daher Gegenstand intensiver Forschungsbemühungen, sowohl in der Radiologie als auch in der Rechtsmedizin. Die radiologische Diagnostik von Pathologien innerhalb des Subduralraums spielt dabei eine zunehmend wichtigere Rolle. In den letzten Jahren wurden mehrere Studien zur diagnostischen und forensischen Relevanz verletzter Brückenvenen mit verschiedenen Bildgebungsmodalitäten durchgeführt. In der Zusammenschau dieser Studien ergeben sich immer deutlichere Belege dafür, dass akute Verletzungen von Brückenvenen und damit im weiteren Verlauf u. U. assoziierte Brückenvenenthrombosen bei lebenden Kindern detektierbar und als Indikatoren einer Kindesmisshandlung zweckdienlich sein können.

## Hintergrund

**Figure 1:**
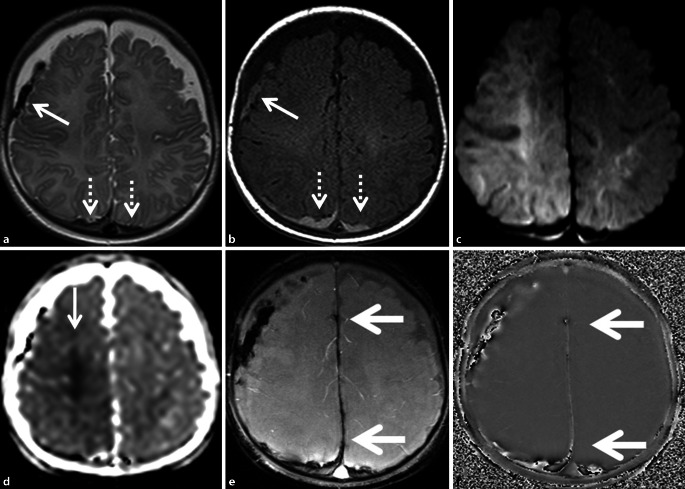

Beim SBS bzw. AHT handelt es sich um eine besonders schwere Form der Kindesmisshandlung, in deren Folge es zu schweren Beeinträchtigungen bis zum Tod des geschädigten Kindes kommen kann. Betroffen sind Säuglinge und Kleinkinder mit einer Häufung zwischen dem 3. und 6. Lebensmonat. Ursache ist vor allem ein gewaltsames Schütteln des zumeist an Oberarmen oder Brustkorb festgehaltenen Kindes [1–3]. Subdurale Flüssigkeitsansammlungen – im internationalen Sprachgebrauch häufig verkürzt als „subdural collections“ (SDC) bezeichnet – stellen dabei die häufigsten Indikatoren des SBS/AHT dar [4–6]. Dabei handelt es sich um verschiedene, sich z. T. stadienartig entwickelnde Befunde innerhalb des unter physiologischen Bedingungen nichtexistenten Subduralraums, insbesondere subdurale Hämatome, Hygrome und Hämatohygrome [7, 8]. Prognosebestimmend ist allerdings in der Regel nicht das Ausmaß der SDC, sondern der Grad der traumatischen Enzephalopathie als Ausdruck der gleichzeitigen Parenchymschädigung infolge von Scherverletzungen mit möglichen Einblutungen und möglicher Sekundärschädigung durch Hypoxie [2, 3]. Ein typisches Fallbeispiel zeigt Abb. 1.

Verletzungen von Brückenvenen, kleinen Meningealgefäßen und der Arachnoidea werden als die wesentlichen Quellen der SDC betrachtet, weshalb SDC vor allem aus Blut bzw. Blutprodukten sowie Liquor und/oder liquorähnlicher Flüssigkeit bestehen [6, 9, 10]. Bei den Brückenvenen handelt es sich um bis zu 50 überwiegend im Subarachnoidalraum verlaufende, im Durchmesser zwischen 0,05 und 3,07 mm große Venen, welche die Kortikalvenen der Oberflächen von Groß- und Kleinhirn mit den großen venösen Sinus verbinden und dabei den inneren Anteil der Dura mater penetrieren [11]. Gruppen von Brückenvenen können vor allem frontal, parietal, temporal und zerebellär gefunden werden [12, 13]. Daneben kommen aber auch anatomische Abweichungen vor.

**Figure 2:**
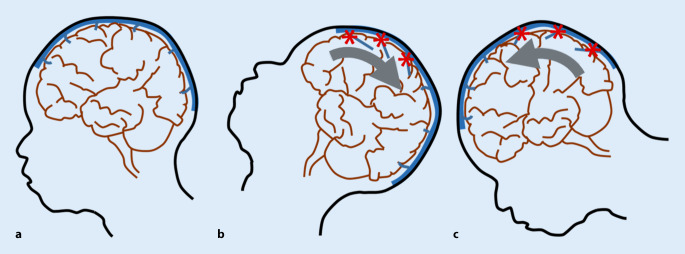

Verletzungen der Brückenvenen, beispielsweise infolge der erheblichen Scher- und Rotationskräfte, wie sie im Rahmen eines Schütteltraumas auftreten (Abb. 2), resultieren typischerweise in extraaxialen Blutungen in den Subarachnoidal- und Subduralraum [11, 14–17]. Neben der pathologischen Eröffnung eines Subduralraums mit Entwicklung subduraler Hämatome kann das venöse Blut auch zur lokalen Thrombosierung innerhalb oder in naher Umgebung von Brückenvenenverletzungen (BVV) führen. Solche Brückenvenenthrombosen (BVT) können von hohem diagnostischem Wert zum Nachweis der traumatischen Natur von SDC im Kontext mit SBS/AHT betrachtet werden [13].

Hinsichtlich der radiologischen Darstellung von BVV und BVT bei lebenden Säuglingen und Kleinkindern hat es in den letzten Jahren einen regen Wissenszuwachs gegeben. BVV wurden dabei zunächst vor allem unter postmortalen Experimentalbedingungen beschrieben [18–23]. Problematisch hierbei ist jedoch, dass die im Rahmen des Obduktionsbetriebs üblicherweise angewandten Techniken zur Entnahme des Gehirns oft artifizielle BVV verursachen [19, 24]. Die retrospektive Untersuchung vitaler, also zu Lebzeiten entstandener, BVV am toten Gehirn erfordert daher spezielle aufwändigere Entnahmetechniken bzw. apparative postmortal-angiographische Technologien und Protokolle [19, 22, 25], die in vielen Institutionen nicht immer oder gar nicht zur Verfügung stehen.

Eine andere Situation besteht bei Lebenden im klinischen Kontext. Der vorliegende Beitrag bietet eine Übersicht über die aktuelle Studienlage zur pädiatrischen Radiologie der BVV und BVT. Dabei wird der aktuelle Forschungsstand dargestellt, und forensische Implikationen werden erörtert.

## Studienlage

Nach selektiver Literaturrecherche mittels einschlägiger bibliografischer Datenbanken (MEDLINE, Cochrane, SCOPUS, EMBASE, Google Scholar) konnten insgesamt 7 Publikationen identifiziert werden, die eine radiologische Untersuchung von BVV und/oder BVT bei lebenden Säuglingen und Kleinkindern beinhalteten. Eine Zusammenfassung der wesentlichen inhaltlichen Parameter dieser Arbeiten zeigt Tab. 1.

**Table 1:** 

Publikation	Fälle mit AHT	Altersbereich	Fälle mit BVV	Fälle mit BVT	Verwendete Untersuchungsmodalität(en)
Barlow et al. (1999) [26]	15	1–34 Mo.	4(?)	n.e.	MRT
Adamsbaum/Rambaud (2012) [27]	3	4–7 Mo.	3	3	CT + MRT
Yilmaz et al. (2014) [28]	2	10 Wo. + 6 Mo.	2	2	MRT
Hahnemann et al. (2015) [29]	29	0–2 J.	11	11	CT + MRT + MR‑V
Choudhary et al. (2015) [30]	45	15 T. bis 3 Mo.	20	20	MRT + MR‑V
Zuccoli et al. (2017) [31]	17	n.e.^a^	14	4	MRT
Ronning et al. (2018) [32]	55	n.e.^b^	45	45	CT

*T.* Tage; *Mo.* Monate; *Wo.* Wochen; *J.* Jahre; *n.e.* nicht erwähnt

*AHT *„abusive head trauma“, *MRT* Magnetresonanztomographie, *CT* Computertomographie, *MR‑V* Magnetresonanzvenographie

^a^nur Interquartilsbereich angegeben: 4–13 Mo.

^b^nur Interquartilsbereich angegeben: 5 T. bis 11,3 Mo.

Barlow et al. (1999; [26]) erwähnten erstmals BVV bei SBS/AHT in der modernen radiologischen Schnittbildgebung. Die Autoren untersuchten alle 15 Kinder, die zwischen 1995 und 1998 im *Royal Hospital for Sick Children, Edinburgh (UK),* mit der Diagnose AHT behandelt wurden. In 8 Fällen hätten Tatgeständnisse vorgelegen, wobei u. a. auch das Schütteln des Kindes nach einem behaupteten Anfallsereignis als Geständnis gewertet wurde. Die Kinder waren zwischen 1 und 34 Monate alt (Durchschnittsalter: 5,7 Monate). Zwölf der 15 Fälle wurden unmittelbar in der Akutphase mittels MRT untersucht. Bei 2 dieser 12 Fälle fand eine wiederholte MRT-Untersuchung noch in der Akutsituation statt; daher waren es insgesamt 14 MRT-Untersuchungen. BVV werden in 4 der 14 MRT-Untersuchungen als „evidence of tearing of the surface veins“ benannt. Auf einer Abbildung eines axialen Gradientenechoschnittbilds werden drei temporoparietal gelegene, dem Kortex dicht anliegende, rundlich bis ovalär gestaltete, hypointense Strukturen von ca. 1- bis 2‑facher Kortexbreite als BVV identifiziert. Aufgrund der 2 Fälle mit Wiederholungsuntersuchungen bleibt unklar, wie viele AHT-Fälle insgesamt BVV aufwiesen. Sieben der 12 AHT-Fälle mit MRT zeigten keine Hinweise für eine stumpfe Gewalteinwirkung gegen den Kopf und wurden daher als Opfer eines Akzelerations-Dezelerations-Traumas durch Schütteln im Sinne eines SBS betrachtet. Von diesen 7 Fällen wiesen 2 Fälle BVV auf. Auch hier bleibt unklar, ob es sich um einen einzigen Fall mit 2 MRT-Untersuchungen oder tatsächlich um zwei unterschiedliche Fälle handelt.

Adamsbaum und Rambaud wiesen 2012 in einem Kommentar [27] auf die besondere Bedeutung der Untersuchung der Vertexregion zur radiologischen Detektion von BVV und BVT bei Verdachtsfällen von SBS/AHT hin. Jeweils mittels Präsentation von CT- und MRT-Bildmaterial wurden insgesamt 3 AHT-Fälle von Säuglingen im Alter zwischen 4 und 7 Monaten illustriert, die in 2 von 3 Fällen mittels Tatgeständnissen verifiziert wurden und sowohl subdurale Hämatome als auch BVT zeigten. Insbesondere die für Blutabbauprodukte sensitive T2*-Sequenz erwies sich dabei als hilfreich beim Erkennen der BVT. Die Autoren beschrieben die BVT als Gerinnsel mit tubulärer Form („clots with tubular shape“), welche als Marker akut verletzter Brückenvenen und damit als Beleg für die traumatische Natur der subduralen Hämatome betrachtet werden sollten. Abschließend wurde diskutiert, dass diese tubulären Gerinnsel auch geronnenem Blut im Subarachnoidalraum entsprechen könnten.

Yilmaz et al. (2014; [28]) beschrieben 2 zum Zeitpunkt der Publikation strafrechtlich noch nicht abschließend bearbeitete AHT-Verdachtsfälle im Alter von 10 Wochen und 6 Monaten mit subduralen Hämatomen und multifokalem tubulusförmigem Signalverlust an Brückenvenen in der für Blutprodukte wie Desoxyhämoglobin, Methämoglobin, Hämosiderin sowie Kalzium- und Eisenablagerungen besonders sensitiven suszeptibilitätsgewichteten MRT-Bildgebung (SWI [„susceptibility-weighted imaging“]). Diese Signalverluste wurden als Gerinnselbildungen verletzter Brückenvenen interpretiert. Hinsichtlich der in beiden Fällen fehlenden Signalveränderungen in der diffusionsgewichteten MRT-Bildgebung (DWI [„diffusion-weighted imaging“]) bzw. hinsichtlich des Fehlens von Zeichen venöser Infarzierung wurde diskutiert, dass dies möglicherweise auf eine inkomplette Rupturierung der Brückenvenen mit persistierender Drainage und teilweiser Gerinnsellokalisation an den Außenseiten geschlossener Venenrupturareale erklärt werden könnte.

**Figure 3:**
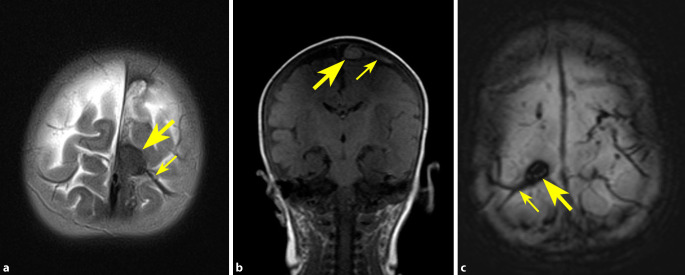

Hahnemann et al. (2015; [29]) untersuchten 29 Kinder der Altersgruppe 0–2 Jahre, die zwischen 2002 und 2013 im Rahmen von CT- und/oder MRT-Untersuchungen (einschließlich MR-Venographie) am Universitätsklinikum Essen mit subduralen Hämatomen und/oder subduralen Hygromen aufgefallen waren. Elf dieser Fälle (40 %, Durchschnittsalter: 5,0 Monate) zeigten vertexnahe gefäßförmige Strukturen, die den früheren Beschreibungen von BVT entsprachen [26–28]. Diese stellten sich ausschließlich in beiden Parasagittalregionen der Hirnkonvexitäten der Frontal- und Parietallappen dar und imponierten in 8 der 11 Fälle als Kaulquappen-ähnliche Strukturen mit am ehesten subarachnoidal gelegenen, rundlich bis oval geformten Thrombusformationen („Körper der Kaulquappe“) und damit assoziierten, verletzt bzw. thrombotisch erweitert wirkenden Brückenvenen („Schwanz der Kaulquappe“). Für diese Befunde wurde der Begriff „tadpole sign“ (Kaulquappen-Zeichen) vorgeschlagen (Abb. 3). Tadpole-sign-Formationen konnten dabei am besten mittels T1-gewichteter Spin-Echo-Sequenz und T2*- bzw. SWI-Sequenz dargestellt werden. Eine zusätzliche Befunderhebung (Netzhautblutungen, Verletzungen von Skelett und Haut), einschließlich umfassender rechtsmedizinischer Begutachtung in 5 der 11 Fälle, ergab, dass alle 11 Fälle mit sehr hoher Wahrscheinlichkeit der Diagnose SBS/AHT zuzuordnen waren. Abschließend wurde geschlussfolgert, dass BVT bei Ausschluss akzidenteller Traumatisierung einen starken Indikator für SBS/AHT darstellen und dass bei Feststellung von BVT bzw. dem „tadpole sign“ unbedingt Verdacht geschöpft bzw. nach weiteren Anzeichen eines SBS/AHT gesucht werden sollte.

Ebenfalls im Jahr 2015 berichteten Choudhary et al. [30] über eine US-amerikanische Studienkohorte von 45 Kindern im Alter von 15 Tagen bis 31 Monaten (Median: 3 Monate), die zwischen 2001 und 2012 die Diagnose AHT erhielten und mittels MRT und MR-Venographie untersucht wurden. In 20 Fällen (44 %) wurden abrupt an subarachnoidalen Blutgerinnseln endende, parasagittale Brückenvenen in Regionen mit darüber befindlichen Subduralhämatomen gefunden. Diese Formationen wurden als direkte BVV mit nachfolgender posttraumatischer Thrombosierung interpretiert und mit dem Begriff „lollipop sign“ bezeichnet. Die Identifizierung gelang dabei am besten unter Betrachtung konventioneller MRT-Sequenzen, insbesondere mittels Gradientenechosequenz (GRE). Ein weiterer Schwerpunkt der Arbeit lag in der Untersuchung von Masseneffekten auf die venösen Sinus und Kortikalvenen. Abschließend wurde auf die Wichtigkeit des Verständnisses von (kompressionsbedingten) Effekten subduraler Hämatome und Hirnschwellung auf kortikale Venen und Sinus hingewiesen, um diese von Kortikalvenen- und Sinusvenenthrombosen abzugrenzen.

Zuccoli et al. (2017) [31] untersuchten den Nutzen einer hochauflösenden koronaren SWI-Sequenz für die Darstellung von BVV/BVT bei 17 Kindern, die zwischen 2012 und 2015 am *Children’s Hospital of Pittsburgh (USA)* die Diagnose AHT erhielten und ein medianes Alter von 8 Monaten aufwiesen (Interquartilsbereich: 4 bis 13 Monate). In 11 Fällen (65 %) wurden mögliche BVT in der axialen Standard-SWI-Sequenz festgestellt, von denen 5 (29 %) ein „tadpole sign“ und 6 (35 %) eine BVT ohne „tadpole sign“ zeigten. In nur 4 von 11 BVT-Verdachtsfällen (36 %) konnte das Vorhandensein einer thrombosierten Vene mittels hochauflösender koronarer SWI-Sequenz bestätigt werden. Anders als in den beiden vorangegangenen Studien wurden BVT somit nur bei 24 % (4 von 17 Fällen) der hier untersuchten Studienkohorte gefunden. Darüber hinaus wurden in 14 Fällen (82 %) mittels hochauflösender koronarer SWI-Sequenz „Irregularitäten“ an den Gefäßwänden der Brückenvenen festgestellt, welche als Brückenvenenrupturen (also BVV) interpretiert wurden und signifikant mit subduralen Hämatomen assoziierten waren (*p* = 0,03). Die Autoren diskutierten, dass ein Großteil derjenigen Patienten mit traumatischem „tadpole sign“ nicht Patienten mit BVT, sondern in Wahrheit Patienten mit („nur“) rupturierten Brückenvenen sein könnten und daher die bislang berichtete Inzidenz von BVT bei SBS/AHT möglicherweise überschätzt worden ist.

Ronning et al. [32] analysierten 99 Säuglinge mit subduralen Hämatomen hinsichtlich des Auftretens von parasagittalen und im Scheitelbereich gelegenen Gerinnselbildungen („parasagittal vertex clots“, PSVC = BVT) im kranialen Computertomogramm. Alle Patienten waren unter 12 Monate alt (mittleres Alter: 4 Monate) und wurden zwischen 2004 und 2014 am *Children’s Hospital and Clinics of Minnesota (USA) *behandelt: 55 Fälle wurden einem AHT zugeordnet (Gruppe 1), 22 Fälle waren zumindest verdächtig auf ein AHT (Gruppe 2) und bei den verbliebenen 22 Fällen (Gruppe 3) wurde keine Misshandlung festgestellt (unfallbedingtes Trauma in 20 Fällen und perinatales Subduralhämatom in 2 Fällen). In der AHT-Gruppe (1) wurden in 45 Fällen (81,2 %) PSVC festgestellt, in der Nicht-AHT-Gruppe (3) hingegen nur in 8 Fällen (36,4 %). Verglichen mit den Patienten ohne PSVC waren die PSVC-Patienten signifikant wahrscheinlicher in der Gruppe mit AHT zu finden (66,2 % vs. 35,5 %; *p* = 0,001). Für PSVC bei AHT in der CT wurden eine Sensitivität von 0,82 (95 % Konfidenzintervall [KI] 0,69–0,90), eine Spezifität von 0,64 (95 % KI 0,41–0,82), ein positiver prädiktiver Wert von 0,85 (95 % KI 0,72–0,93) und ein negativer prädiktiver Wert von 0,59 (95 % KI 0,37–0,77) angegeben. In einer zusätzlichen Untersuchung von 87 CT-Scans von Säuglingen mit „bekanntem akzidentellem Trauma“ und ohne Subduralhämatom (Gruppe 4) fanden sich keine PSVC.

## Diskussion

Im Jahr 2018 wurde ein umfassendes „Consensus Statement“ zum AHT publiziert [33], welches von einer Vielzahl namhafter medizinischer Fachgesellschaften aus der ganzen Welt (einschließlich der deutschsprachigen Gesellschaft für Pädiatrische Radiologie) unterstützt wird [34]. Zur Reduktion von Unklarheiten wird darin u. a. der derzeitig empfohlene Diagnostikprozess dezidiert dargestellt und darüber hinaus auch spekulativen Alternativtheorien zur Pathogenese und Biomechanik des SBS/AHT entgegengetreten, wie sie nach wie vor insbesondere in Gerichtsprozessen – zumeist vonseiten der Verteidigung – vorgebracht werden.

Die Erfahrung vor Gericht zeigt, dass die Biomechanik und Anatomie von BVV im Rahmen von SBS/AHT-Ereignissen bei solchen Alternativtheorien nicht selten im Fokus von Argumentationen stehen, wenngleich hierbei – zumindest hinsichtlich der morphologischen Tatsachen – vergleichsweise wenig Raum für Spekulationsmöglichkeiten besteht. Schon länger ist bekannt, dass Brückenvenen an unterschiedlichen Stellen über unterschiedliche Wandstärken verfügen. Während die Brückenvenenwand im Subarachnoidalraum zwischen 50 und 200 µm misst, kann sie in dem Bereich, in welchem die Brückenvene durch die Dura mater tritt, eine Breite von nur noch 10 µm aufweisen [35]. Auch die im Subarachnoidalraum noch bestehende zusätzliche Wandverstärkung durch umliegendes Bindegewebe findet sich in dem dünnwandigeren Bereich nicht mehr [35]. Aus diesen Gründen wird eine erhöhte Vulnerabilität vornehmlich der duralen Brückenvenenabschnitte angenommen [35]. Die bei Verletzung der Brückenvene resultierende Blutung ermöglicht daher erst die Eröffnung eines subduralen Kompartiments (Subduralraum), das streng genommen aktuell eher als eine intradurale Läsion durch Auf- oder Abspaltung des innersten Anteils der Dura mater – der sog. duralen Grenzzellschicht („dural border cell layer“), also der der Arachnoidea am nächsten gelegenen duralen Zellschicht – betrachtet wird [6, 36, 37]. Dieser pathologische Raum besteht unter physiologischen Umständen nicht. Dennoch ist der Begriff „subdural“ nach wie vor weitgehend üblich, sodass eine Blutung aus einer oder mehreren Brückenvenen zu dem Phänomen führt, was mithin als Subduralhämatom bezeichnet wird.

Hinsichtlich des radiologischen Beitrags zur Diagnostik des SBS/AHT betonten Orman et al. [38] kürzlich, dass neben dem bei SBS/AHT häufigsten neuroradiologischen Befund des Subduralhämatoms auch die sog. „weniger bekannten MRT-Befunde“ berücksichtigt werden sollten. Hierbei waren Befunde gemeint wie parenchymale oder kortikale Lazerationen, subpiale Blutungen, Verletzungen des kraniozervikalen Übergangs einschließlich retroclivaler Hämatome, sog. „diffuse“ hypoxische Hirnschäden oder auch das „tadpole sign“ [29] bzw. „lollipop sign“[30].

Da ein Lollipop-Zeichen bereits in der Literatur als radiologisches Merkmal bei der Diagnostik des hepatischen epithelioiden Hämangioendothelioms beschrieben wurde [39], wäre vorzuschlagen, im Rahmen der SBS/AHT-Thematik eher den Begriff „tadpole sign“ zu verwenden. Dennoch dürfte davon auszugehen sein, dass das „tadpole sign“ und das (AHT-assoziierte) „lollipop sign“ grundsätzlich dieselben Strukturen beschreiben. Die Mehrzahl der oben näher betrachteten Studien [27–30, 32] interpretierten sie als BVT. Diese Interpretation als verletzte und thrombosierte Brückenvenen wird auch durch verschiedene autoptische Untersuchungen untermauert, beispielsweise von Rambaud (2015; [13]).

Die Arbeit von Zuccoli et al. [31] stellte diese Interpretation erstmals infrage, da nur in 4 von 11 BVT-Verdachtsfällen (36 %) das Vorhandensein einer thrombosierten Vene mittels hochauflösender koronarer SWI-Sequenz bestätigt worden sei. Unabhängig jedoch von der Frage, ob es sich beim „tadpole sign“ um eine Ausprägung von BVT handelt oder nicht, so stellten Zuccoli et al. [31] dennoch in 82 % der Fälle „Irregularitäten“ an den Gefäßwänden der Brückenvenen fest, welche sie als Brückenvenenrupturen (also BVV) interpretierten. Die zusätzlich bestehende Interpretationsmöglichkeit von Rekanalisierungen ehemals intraluminaler Thromben wird von den Autoren nicht diskutiert. Die Autoren vermuteten jedoch, dass ein Großteil derjenigen Patienten mit „tadpole sign“ nicht Patienten mit BVT, sondern in Wahrheit Patienten mit (*nur*) rupturierten Brückenvenen sein könnten. Die traumatische Natur des „tadpole sign“ bliebe somit auch bei kritischer Betrachtung bestehen. Dies unterstützt wiederum die Annahme, dass das „tadpole sign“, obgleich es nach Zuccoli et al. [31] möglicherweise seltener mit (zumindest vollständig lumenverschließenden) BVT assoziiert sein könnte als zuvor angenommen, trotzdem einen charakteristischen und richtungsweisenden traumatischen Befund darstellt und damit die Verdachtsdiagnose eines stattgehabten SBS/AHT erhärtet.

Ursache für die wie BVT imponierenden Formationen könnte nach Ansicht von Zuccholi et al. [31] ein Partialvolumeneffekt sein, wodurch rupturierte Brückenvenen und Hämosiderinablagerungen in der axialen Standard-T2*-GRE-Sequenz mit 5 mm Schichtdicke wie BVT imponieren. Eine anatomisch hochauflösende 3‑D-SWI mit Isovoxel-Akquisition wäre hier nach unserer Ansicht hilfreich, um das „tadpole sign“ zu verifizieren.

Die Frage, ob sich BVT oder das „tadpole sign“ auch zu altersdiagnostischen Zwecken eignen, ist noch nicht abschließend geklärt. Die Neuropathologin Rambaud [13] wies darauf hin, dass es naturgemäß mitunter mehrere Stunden dauern kann, bis rupturierte Brückenvenen einen (intraluminalen) Thrombus ausbilden, falls dies überhaupt eintritt. Auch dieser Prozess hängt bekanntermaßen von verschiedenen Einflussfaktoren wie dem Durchmesser der Brückenvene bzw. der Größe der Rupturstelle (ganzes Gefäß oder *nur* Einriss) oder der allgemeinen Gerinnungssituation des Organismus ab. Ob BVT oder „tadpole sign“ somit als Hinweis für eine beispielsweise akute oder (früh) subakute Traumasituation interpretiert werden können, ließe sich nur anhand weiterer und größerer Fallkollektive unter Einschluss auch von fallbezogenen Verlaufskontrolluntersuchungen zweifelsfrei belegen.

Um die beschriebenen Zeichen der BVV und BVT nachzuweisen, bedarf es allerdings einer dezidierten, artefaktfreien (bzw. zumindest artefaktarmen) MR-Technik und sorgfältiger multiplaner Rekonstruktionen. Während mit den Standard-T2*-GRE-Techniken zwar Suszeptibilitätsartefakte und damit Blutungen bzw. Thromben gut verifiziert werden können, ist für die o. g. Zeichen eine höhere Ortsauflösung und spezifischere Bildgebung notwendig, wie sie die hochauflösende SWI bietet. In der aktuellen S3-Kinderschutzleitlinie [40] wird die SWI bereits erwähnt, aufgrund der (noch) zu geringen Evidenz allerdings nicht generell empfohlen.

Für die Praxis radiologischer Gutachtertätigkeit ist festzuhalten, dass BVV bzw. BVT und das „tadpole sign“ die verletzungsbedingte Natur von subduralen Hämatomen zwar belegen, *bei alleiniger Betrachtung* jedoch nicht als Beleg für oder gegen eine stattgehabte Kindesmisshandlung herangezogen werden können. Bereits Hahnemann et al. [29] stellten fest, dass BVT nur bei Ausschluss akzidenteller Traumatisierung einen starken Indikator für SBS/AHT darstellen. Ronning et al. [32] konnten außerdem durch Feststellung von BVT in 36,4 % der Fälle einer „Nicht-AHT-Gruppe“ mit subduralen Hämatomen explizit aufzeigen, dass BVT nicht AHT-spezifisch sind. Somit muss – wie in rechtsmedizinischen Begutachtungsfällen von Kindesmisshandlung üblich – auch für die Bestätigung der Verdachtsdiagnose „SBS/AHT“ stets das Gesamtbild *aller* festgestellten Verletzungen betrachtet werden. Das oft von Gegnern des Schütteltrauma-Konzepts praktizierte selektive Herausgreifen einzelner AHT-typischer Verletzungsbefunde (wie Netzhautblutungen, subdurale Hämatome oder eben BVT) führt nahezu immer auch zur Auflistung akzidenteller oder krankheitsbedingter Alternativursachen, was jedoch nicht den Wert dieser Befunde für die SBS/AHT-Diagnostik mindert. Daher sind auch BVV/BVT und „tadpole sign“ nach wie vor als wertvolle Indikatoren einer potenziell lebensbedrohlichen Kindesmisshandlung zu empfehlen.

## Fazit für die Praxis

- Bei Verdachtsfällen von SBS/AHT („shaken baby syndrome“/„abusive head trauma“) empfiehlt sich im Rahmen von CT- und MRT-Untersuchungen des Kopfes die Suche nach vertexnah gelegenen Brückenvenenverletzungen, da hierdurch die traumatische Natur von Subduralhämatomen belegt werden kann.
- Brückenvenenverletzungen imponieren häufig als rundlich-erweitert wirkende bzw. tubulär gestaltete Strukturen mit/ohne Thrombose, wobei das „tadpole sign“ als wertvolles Hilfsmittel zu deren Identifizierung dienen kann.
- Für die CT-Diagnostik sind koronare Rekonstruktionen zusätzlich zu den axialen Primäraufnahmen erforderlich, um die vertexnahen Befunde zuverlässig zu detektieren.
- Insbesondere T2*/SWI-Sequenzen ermöglichen eine gute Detektion von Brückenvenenverletzungen und sollten bei V. a. SBS/AHT immer miterstellt werden, wobei anatomisch hochauflösenden SWI der Vorzug gegeben werden sollte.
- Das Vorhandensein von radiologisch detektierbaren Brückenvenenverletzungen sollte stets Anlass dazu geben, nach weiteren Anzeichen einer möglichen Kindesmisshandlung zu suchen.

## References

[1] Christian CW, Block R, Committee on Child Abuse and Neglect, American Academy of Pediatrics (2009). Abusive head trauma in infants and children. Pediatrics.

[2] Matschke J, Herrmann B, Sperhake J, Körber F, Bajanowski T, Glatzel M (2009). Shaken baby syndrome: a common variant of non-accidental head injury in infants. Dtsch Arztebl Int.

[3] Herrmann B, Dettmeyer R, Banaschak S, Thyen U, Herrmann B, Dettmeyer R, Banaschak S, Thyen U (2016). Misshandlungsbedingte Kopfverletzungen und Schütteltrauma-Syndrom. Kindesmisshandlung – Medizinische Diagnostik, Intervention und rechtliche Grundlagen.

[4] Hymel KP, Jenny C, Block RW (2002). Intracranial hemorrhage and rebleeding in suspected victims of abusive head trauma: addressing the forensic controversies. Child Maltreat.

[5] Vinchon M, de Foort-Dhellemmes S, Desurmont M, Delestret I (2010). Confessed abuse versus witnessed accidents in infants: comparison of clinical, radiological, and ophthalmological data in corroborated cases. Childs Nerv Syst.

[6] Hedlund GL, Kleinman PK (2016). Abusive head trauma: extra-axial hemorrhage and nonhemic collections. Diagnostic imaging of child abuse.

[7] Wittschieber D, Karger B, Niederstadt T, Pfeiffer H, Hahnemann ML (2015). Subdural hygromas in abusive head trauma: pathogenesis, diagnosis, and forensic implications. AJNR Am J Neuroradiol.

[8] Wittschieber D, Karger B, Pfeiffer H, Hahnemann ML (2019). Understanding subdural collections in pediatric abusive head trauma. AJNR Am J Neuroradiol.

[9] Osborn AG, Osborn AG (2013). Trauma. Osborn’s brain: imaging, pathology, and anatomy.

[10] Wittschieber D, Kinner S, Pfeiffer H, Karger B, Hahnemann ML (2018). Forensische Aspekte bildgebender Verfahren bei Schütteltrauma-Syndrom: Methodik, Befunde, Differenzialdiagnosen. Rechtsmedizin.

[11] Cheshire EC, Malcomson RDG, Sun P, Mirkes EM, Amoroso JM, Rutty GN (2018). A systematic autopsy survey of human infant bridging veins. Int J Legal Med.

[12] Mortazavi MM, Denning M, Yalcin B, Shoja MM, Loukas M, Tubbs RS (2013). The intracranial bridging veins: a comprehensive review of their history, anatomy, histology, pathology, and neurosurgical implications. Childs Nerv Syst.

[13] Rambaud C (2015). Bridging veins and autopsy findings in abusive head trauma. Pediatr Radiol.

[14] Case ME, Graham MA, Handy TC, Jentzen JM, Monteleone JA, National Association of Medical Examiners Ad Hoc Committee on Shaken Baby Syndrome (2001). Position paper on fatal abusive head injuries in infants and young children. Am J Forensic Med Pathol.

[15] Nierenberger M, Wolfram-Gabel R, Decock-Catrin S, Boehm N, Rémond Y, Kahn JL, Ahzi S (2013). Investigation of the human bridging veins structure using optical microscopy. Surg Radiol Anat.

[16] Minns RA (2005). Subdural haemorrhages, haematomas, and effusions in infancy. Arch Dis Child.

[17] Morrison CN, Minns RA, Minns RA, Brown JK (2006). The biomechanics of shaking. Shaking and other non-accidental head injuries in children.

[18] Norman MG, Smialek JE, Newman DE, Horembala EJ (1984). The postmortem examination on the abused child. Pathological, radiographic, and legal aspects. Perspect Pediatr Pathol.

[19] Maxeiner H (1997). Detection of ruptured cerebral bridging veins at autopsy. Forensic Sci Int.

[20] Maxeiner H (2001). Demonstration and interpretation of bridging vein ruptures in cases of infantile subdural bleedings. J Forensic Sci.

[21] Ehrlich E, Maxeiner H, Lange J (2003). Postmortem radiological investigation of bridging vein ruptures. Leg Med.

[22] Stein KM, Ruf K, Ganten MK, Mattern R (2006). Representation of cerebral bridging veins in infants by postmortem computed tomography. Forensic Sci Int.

[23] Depreitere B, Van Lierde C, Sloten JV (2006). Mechanics of acute subdural hematomas resulting from bridging vein rupture. J Neurosurg.

[24] Squier W, Mack J (2009). The neuropathology of infant subdural haemorrhage. Forensic Sci Int.

[25] Chevallier C, Michaud K, Palmiere C, Alamo L, Mangin P, Grabherr S (2015). Multiphase postmortem computed tomography angiography in pediatrics: a case report. Am J Forensic Med Pathol.

[26] Barlow KM, Gibson RJ, McPhillips M, Minns RA (1999). Magnetic resonance imaging in acute non-accidental head injury. Acta Paediatr.

[27] Adamsbaum C, Rambaud C (2012). Abusive head trauma: don’t overlook bridging vein thrombosis. Pediatr Radiol.

[28] Yilmaz U, Körner H, Meyer S, Reith W (2015). Multifocal signal loss at bridging veins on susceptibility-weighted imaging in abusive head trauma. Clin Neuroradiol.

[29] Hahnemann ML, Kinner S, Schweiger B, Bajanowski T, Karger B, Pfeiffer H, Wittschieber D (2015). Imaging of bridging vein thrombosis in infants with abusive head trauma: the “Tadpole Sign”. Eur Radiol.

[30] Choudhary AK, Bradford R, Dias MS, Thamburaj K, Boal DK (2015). Venous injury in abusive head trauma. Pediatr Radiol.

[31] Zuccoli G, Khan AS, Panigrahy A, Tamber MS (2017). In vivo demonstration of traumatic rupture of the bridging veins in abusive head trauma. Pediatr Neurol.

[32] Ronning MM, Carolan PL, Cutler GJ, Patterson RJ (2018). Parasagittal vertex clots on head CT in infants with subdural hemorrhage as a predictor for abusive head trauma. Pediatr Radiol.

[33] Choudhary AK, Servaes S, Slovis TL, Palusci VJ, Hedlund GL, Narang SK, Moreno JA, Dias MS, Christian CW, Nelson MD, Silvera VM, Palasis S, Raissaki M, Rossi A, Offiah AC (2018). Consensus statement on abusive head trauma in infants and young children. Pediatr Radiol.

[34] Choudhary AK, Narang SK, Moreno JA, Christian CW, Servaes S, Palusci VJ, Hedlund GL, Dias MS, Nelson MD, Silvera VM, Palasis S, Raissaki M, Rossi A, Offiah AC (2019). A consensus response on the complete picture: reply to Lynøe and Eriksson. Pediatr Radiol.

[35] Yamashima T, Friede RL (1984). Why do bridging veins rupture into the virtual subdural space?. J Neurol Neurosurg Psychiatry.

[36] v. Düring M, Dermietzel R, Drenckhahn D, Benninghoff A, Drenckhahn D (2004). Hirnhäute, Ventrikelauskleidung, Liquor cerebrospinalis. Anatomie.

[37] Mack J, Squier W, Eastman JT (2009). Anatomy and development of the meninges: implications for subdural collections and CSF circulation. Pediatr Radiol.

[38] Orman G, Kralik SF, Meoded A, Desai N, Risen S, Huisman TAGM (2020). MRI findings in pediatric abusive head trauma: a review. J Neuroimaging.

[39] Alomari AI (2006). The lollipop sign: a new cross-sectional sign of hepatic epithelioid hemangioendothelioma. Eur J Radiol.

[40] AWMF (2019) S3-Leitlinie „Kindesmisshandlung, -missbrauch, -vernachlässigung unter Einbindung der Jugendhilfe und Pädagogik (Kinderschutzleitlinie)“. https://www.awmf.org/uploads/tx_szleitlinien/027-069l_S3_Kindesmisshandlung-missbrauch-vernachlaessigung-Kinderschutzleitlinie_2019-02_1_01.pdf. Zugegriffen: 29.11.2020 10.1007/s00104-020-01270-z 32974787

